# Bionic Bovine Achilles Tendon Collagen Composite Membrane Loaded with Anti-Inflammatory Kukoamine B Promotes Skin Wound Healing

**DOI:** 10.3390/polym17131874

**Published:** 2025-07-04

**Authors:** Ruting Luo, Yujie Mu, Le Zhao, Jinglin Hua, Lixin Cao, Danting Chen, Kun Li, Zhenkai Jin, Yanchuan Guo, Bing Zhang, Min Wang

**Affiliations:** 1 School of Light Industry Science and Engineering, Beijing Technology and Business University, Beijing 100048, China; lrt18948322588@163.com (R.L.); muyujie5576@163.com (Y.M.); h15011089268@163.com (J.H.); lixincao_2312@163.com (L.C.); 18873262945@163.com (D.C.); 2 School of Pharmacy, Henan University of Chinese Medicine, Zhengzhou 450046, China; zhaole@hactcm.edu.cn; 3 BTBU-YHZK Bioengineering Raw Material Development Laboratory, Beijing Technology and Business University, Beijing 102401, China; likun_135@126.com (K.L.); jinzhen_kai852@126.com (Z.J.); 4 Technical Institute of Physics and Chemistry CAS, Beijing 100190, China; yanchuan_g@163.com

**Keywords:** Bovine Achilles Tendon Collagen, dressing, electrospun, anti-inflammatory, wound healing

## Abstract

Skin is the first line of defence between the human body and the outside world, and it is constantly exposed to external injuries and wounds for a variety of reasons. Collagen is a structural protein of the extracellular matrix and an important component of the dermis. As a wound dressing, collagen not only provides nutrients to wounds but also enhances the immune response in the pre-healing phase, making it an excellent biomaterial for healing. In this study, we used electrospinning and freeze-drying technology to prepare a Bovine Achilles Tendon Collagen (BATC) electrospun composite membrane and a BATC freeze-dried composite membrane using BATC as a substrate supplemented with 16.7% Polyethylene oxide (PEO) and 0.2% Kukoamine B (KuB). The physicochemical properties and biocompatibility of the BATC composite membrane were verified via scanning electron microscopy, Fourier-transform infrared spectroscopy, and DSC analysis and by measuring the DPPH radical-scavenging capacity, water absorption, water retention, in vitro drug release, and extract cytotoxicity. The BATC composite membrane was found to have a significant effect on skin wound healing, especially in the middle stage of healing, in a mouse full-thickness skin injury model. The BATC/PEO/KuB electrospun composite membrane (EBPK) had the best capacity for promoting wound healing and can be used as a wound dressing for in-depth research and development, and KuB, a monomer component with a clear structure and mechanism of action, can be used as a candidate component of composite dressings.

## 1. Introduction

Skin plays an essential role in regulating body temperature, protecting the body against foreign substances, and regulating sensory, immune system, and other important functions [1]. However, the integrity of the skin is constantly threatened by numerous external factors, such as injuries, burns, contusions, cuts, trauma, diseases, and surgical incisions [2]. Wound healing is a complex and dynamic process divided into a haemostatic phase, an inflammatory response phase, a tissue proliferation and granulation phase, and a wound contraction and scar formation phase [3]. An inflammatory response occurs during this process, and if a severe inflammatory response occurs and the infection persists without proper treatment, a chronic condition can develop, lasting for months or even years [2]. Some common chronic wounds are diabetic foot ulcers, pressure ulcers, and venous leg ulcers [4,5]. Chronic and non-healing wound sites, where the subcutaneous tissue of a wound is often exposed to the external environment for long periods of time, predispose patients to bleeding and osteomyelitis, posing a significant risk of death [6]. These wounds are prone to complications such as cellulitis and abscess formation, which can progress to bloodstream infections such as bacteraemia and sepsis if not effectively managed [7]. At the same time, the great duration of this type of wound healing can place a serious psychological burden on patients and their families.

Wound dressings are therefore essential for creating the optimal environment for promoting wound healing and protecting wounds from further injury and infection. The most commonly used wound dressings are natural gauze (e.g., cotton wool and cellulose) and synthetic gauze (e.g., polyamide), which are widely used in wound care and debridement procedures. Gauze is inexpensive and breathable but has limited absorbency, has poor biocompatibility, and lacks antimicrobial properties [3]. As a result, a number of novel materials have been used to develop biomedical dressings for various wound treatments, such as electrospun fibres, hydrogels, and microneedles [8,9,10,11].

Electrospinning is the jet spinning of a polymer solution or melt via the action of a strong electric field [12]. Polyurethane electrospun textiles mimicking the extracellular matrix have good air permeability, high porosity, and considerable drug-carrying capacity, allowing them to provide a good environment for tissue regeneration. They have great potential for use in wound therapy [13]. Electrospun collagen fibres have a structure very close to the ultrastructure of natural tissues, provoking only a limited immune response and offering good biocompatibility, making electrospun collagen fibre scaffolds suitable for most human tissue engineering [14]. Pure collagen fibres have poor mechanical strength, and collagen is usually mixed with synthetic polymers during the preparation of the spinning solution to produce composite fibres. Incorporating other polymers, such as hydroxyapatite, polycaprolactone (PCL), and polyglycolide (PGA), can help improve mechanical properties, reduce degradation rates, and optimise the biological response [15,16].

Collagen, a major component of animal connective tissue and an important component of the human dermis, is widely distributed in living organisms [17,18]. During haemostasis, collagen absorbs water from the blood, concentrates the cellular and protein components of the blood, and slows blood flow. Collagen also causes platelets to aggregate, providing a physical scaffold for platelet clotting, thereby promoting haemostasis. When collagen absorbs water and expands, it can exert pressure on blood vessels to stop bleeding. The inflammatory response lasts from a few hours to about 48 h after injury, and the site of injury is locally red and swollen, with local congestion, plasma exudation, and neutrophil and macrophage excursion. Collagen activates the coagulation response, forming fibrin clots and inhibiting bleeding. Collagen fragments act as chemotactic agents for neutrophils, enhance phagocytosis and immune responses, and regulate gene expression [19].

Kukoamine B (KuB) is a spermidine alkaloid isolated and extracted from the dried root bark of *Lycium chinense* Mill. or *Lycium barbarum* L., family Solanaceae, from the traditional Chinese medicine Cortex Lycii. KuB inhibits the upregulation of the expression of various oncogenes and pro-inflammatory cytokines such as tumour necrosis factor-α, interleukin-6 (IL-6), and inducible nitric oxide synthase and cyclooxygenase-2 [20,21]. In our previous work, we found that KuB had an inhibitory effect on inflammation, so we aim to prepare new biomaterials that can accelerate the healing of skin wounds by loading KuB onto collagen composite membranes.

In this study, we used electrospinning and freeze-drying technology to prepare a BATC electrospun composite membrane and a BATC freeze-dried composite membrane using BATC as a substrate supplemented with 16.7% PEO and 0.2% KuB and investigated the biocompatibility and wound-healing effects of the composite membrane in vitro and in vivo.

The wound-healing process involves a dynamic balance between the modulation of the inflammatory response and tissue regeneration (e.g., fibroblast-mediated collagen deposition). Currently commonly used dressings function mainly by physically isolating the wound but do not sufficiently regulate the inflammatory microenvironment. In this study, we innovatively combined the anti-inflammatory activity of KuB and the biomimetic structure of BATC to achieve synergistic effects of natural drugs and biomaterials by simultaneously regulating the inflammatory microenvironment and promoting collagen deposition. Compared with chemically synthesised drug carriers (e.g., indomethacin-loaded poly(lactic-co-glycolic acid) membranes) [22], collagen membranes loaded with natural components may have the advantage of lower cytotoxicity.

## 2. Materials and Methods

### 2.1. Preparation of the BATC Composite Membrane

BATC electrospun composite membrane: BATC and Polyethylene oxide (PEO) (Jinsui Biotechnology Co., Ltd., Shanghai, China)were dissolved in hexafluoroisopropanol (HFIP) (Aladdin Biochemical Technology Co., Ltd., Shanghai, China), and BATC was mixed with PEO in ratios of 5:1, 10:1, and 20:1 to obtain a spinning solution with a concentration of 5%. A BATC/PEO/KuB electrospun solution was prepared by adding 0.2% KuB (Herbest Co., Ltd., Baoji, China) to the spinning solution in the optimal spinning emergence state. The spinning solution was poured into a syringe, and the corresponding parameters were set as follows: voltage, 20 kv; feed speed, 1 mL/h; spinning needle size, 0.2 mm; receiving distance, 10 cm; roller speed, 300 r/min; and receiving material, aluminium foil. After the spinning process, the electrospun composite membranes were left under a fume hood for 24 h. Thus, a BATC/PEO electrospun composite membrane (EBP) and a BATC/PEO/KuB electrospun composite membrane (EBPK) were prepared.

BATC freeze-dried composite membrane: A total of 0.5 g of BATC was dissolved in 20 mL of 3% acetic acid solution. Dialysis was performed for 4 days, during which time the solution was changed every 12 h. The collagen solution was poured into a large Petri dish and frozen overnight in a −80 °C refrigerator. It was then removed and freeze-dried to produce a BATC freeze-dried membrane (FB). A total of 0.4 g of BATC was dissolved in 20 mL of 3% acetic acid solution, dialysed, supplemented with 0.1 g of PEO, dissolved via magnetic stirring, and freeze-dried to obtain a BATC/PEO freeze-dried composite membrane (FBP). A total of 0.4 g of BATC was dissolved in 20 mL of 3% acetic acid solution. Then, 0.1 g of PEO and 1 mg of KuB were added after dialysis, and all the components were dissolved via magnetic stirring and freeze-dried to obtain a BATC/PEO/KuB freeze-dried composite membrane (FBPK).

### 2.2. Characterisation of the BATC Composite Membrane

#### 2.2.1. Morphology Observations

The microstructures of the prepared samples were observed using scanning electron microscopy (SEM) (S-4800, HITACHI, Tokyo, Japan). The samples were glued to the sample stage with conductive adhesive, and the surface was sprayed with gold and then observed using scanning electron microscopy.

#### 2.2.2. Fourier-Transform Infrared Spectroscopy Spectral Analysis

The functional group compositions of the samples were determined using Fourier-transform infrared spectroscopy (FTIR) (Excalibur 3100, Varian, Palo Alto, CA, USA). Spectral data on the samples were analysed in the ATR mode with a wavenumber range of 600–4000 cm^−1^ for each spectrum.

#### 2.2.3. Thermal Stability Measurement

The denaturation temperatures of the samples were evaluated using a differential scanning calorimeter (214 polyma, Netzsch, Selb, Germany). A total of 3 mg of each sample was extracted and heated from 20 °C to 150 °C using a differential scanning calorimeter at a rate of 5 °C/min.

#### 2.2.4. DPPH Radical-Scavenging Activity

A total of 1 mg of the composite membrane sample was added to 3 mL of 0.1 mmol/L DPPH (Acmec Co., Ltd., Shanghai, China) solution and incubated for 30 min in the dark at room temperature. At the same time, blank and ethanol groups were established, and the absorbance at 517 nm was measured using a microplate reader (Infinite M200 Pro, TECAN, Männedorf, Switzerland). The absorbance was measured again after the samples were incubated in the dark at room temperature for 30 min. Equation (1) was used to obtain the results.

DPPH radical-scavenging activity (%) = (1 − (Z_1_ − Z_2_)/Z_0_) × 100% (1)

where Z_0_ is the absorbance of 0.1 mmol/L DPPH solution at 517 nm, Z_1_ is the absorbance of DPPH solution mixed with the samples, and Z_2_ is the absorbance of anhydrous ethanol mixed with the samples.

#### 2.2.5. Absorbency and Retention Capacity

The samples were dried in an oven at 50 °C for 4 h. The dried samples were then immersed in pure water for 12 h. The water on the surface of the samples was removed, and the changes in the mass of the samples before and after being weighed were determined. Equation (2) was used to calculate the absorbency of the samples.

Absorbency (%) = (M_1_ − M_0_)/M_0_ (2)

where M_0_ is the dry weight of the sample, and M_1_ is the mass of the sample after absorbing water.

The absorbed samples were dried in an oven at 37 °C and weighed every 5 min. Equation (3) was used to calculate the water retention capacity of the samples.

Retention capacity (%) = M_2_/M_1_ × 100% (3)

where M_2_ is the mass of the sample at each time point.

#### 2.2.6. Kukoamine B Release In Vitro

The PBS (Hyclone, Logan, UT, USA) solution containing 0.1 mg/mL of KuB was scanned at full wavelength from 190 to 500 nm using an ultraviolet spectrophotometer (UV-2550, Shimadzu, Kyoto, Japan) to determine the peak wavelength of KuB(A). A 2 mg sample of the composite membrane was placed in 20 mL of PBS and extracted using a thermostatic shaker (37 °C, 80 r/min). A total of 1 mL of the sample solution was collected at 0 h, 0.5 h, 1 h, 2 h, 3 h, 4 h, 5 h, 6 h, 7 h, 8 h, 10 h, and 12 h. The absorbance of the sample solution was measured at wavelength A. Each time a sample solution was collected, 1 mL of PBS was added to ensure that the volume of the system was 20 mL, and then the drug release curve was plotted.

### 2.3. In Vitro Cytotoxicity and Cytocompatibility

#### 2.3.1. Human Skin Fibroblast Cell Culture for In Vitro Models

Human skin fibroblasts (HSFs) were purchased from the National Collection of Authenticated Cells Cultures (Shanghai, China). The HSF cells were cultured in Dulbecco’s modified eagle medium (DMEM) (Life Technologies™ Gibco^®^, Billings, MT, USA) high-sugar medium supplemented with 10% foetal bovine serum (Life Technologies™ Gibco^®^, Billings, MT, USA) and 1% penicillin–streptomycin (Life Technologies™ Gibco^®^, Billings, MT, USA) at 37 °C in a 5% CO_2_ environment.

#### 2.3.2. Preparation of BATC Composite Membrane Extracts

Based on the standard GB/T 1688.12-2017 Biological evaluation of medical devices—Part 12: Sample preparation and reference materials in the preparation specifications [23], the 6 cm^2^ composite membrane samples were sterilised via UV irradiation for 1 h on both sides, supplemented with 1 mL of DMEM, and immersed in a 37 °C incubator for 24 h.

#### 2.3.3. Cytotoxicity Assay

The cytotoxicity of the composite membrane extract on HSF cells was determined using Cell Counting Kit-8 (CCK-8) (Bioss Antibodies Co., Ltd., Beijing, China). The HSF cells were seeded at a density of 5 × 10^3^ cells per well in a 96-well plate and incubated in a 37 °C CO_2_ cell culture incubator for 24 h. The medium was aspirated, and 200 μL of extract was added to each well. After 24 h of incubation, 10 μL of CCK-8 was added to each well and incubated for 2 h in the incubator at 37 °C, protected from light. Finally, the absorbance of each well was detected at 450 nm using a microplate reader to calculate cell viability.

#### 2.3.4. Cytocompatibility Assay

The composite membrane sample was cut into a circle with a diameter of 5 mm and sterilised via UV irradiation for 1 h on both sides. The composite membrane was spread on the bottom of a 96-well plate, and a cloning ring was pressed onto the composite membrane to fix it. HSF cells were seeded into the 96-well plate at a density of 5 × 10^3^ cells per well, and the cell suspension was added to the cloning ring and placed in a 37 °C CO_2_ cell culture incubator. After 24 h of incubation, 10 μL of CCK-8 was added to each well and incubated for 2 h at 37 °C in the incubator, protected from light. Finally, the absorbance of each well was detected at 450 nm using a microplate reader in order to calculate the cell viability.

### 2.4. In Vivo Study of BATC Composite Membrane Designed to Promote Skin Wound Healing

#### 2.4.1. Establishment of a Mouse Wound Model

Full-thickness skin injury models of 6–8-week-old male C57BL/6 mice were randomly divided into an FB group, an FBPK group, an EBP group, an EBPK group, a gauze group, and a commercially available Bovine Achilles Tendon Collagen dressing (CDCD) (Biot Co., Ltd., Wuxi, China) group, with four mice in each group. Before surgery, the mice were anaesthetised, and their backs were shaved, with a shaved area of approximately 2.5 cm × 2.5 cm, and wiped with PBS. A 1 cm diameter circular wound was made on the backs of the mice, using surgical scissors to ensure adequate removal of skin, dermis, and myolemma. A 1.5 cm^2^ specimen was then applied externally to the wound and held in place with medical tape. The dressing was changed on post-operation day 7, and the old dressing was moistened with saline to prevent secondary damage to the wound. Wound healing was observed after surgery, and photographs of the wounds were taken on days 0, 3, 5, 7, 10, and 14 with the aid of a ruler and analysed using Image-J 1.44 software. Equation (4) was used to calculate the wound-healing rate.

Healing rate (%) = (original wound area − healed wound area)/original wound area × 100%(4)

#### 2.4.2. Histological and Immunohistochemical Analysis

Skin tissues were collected from the wound at low temperature on days 7 and 14, fixed overnight in 10% neutral formaldehyde solution, and embedded in paraffin. Subsequently, H&E staining (Servicebio Technology Co., Ltd., Wuhan, China) and Masson staining (Servicebio Technology Co., Ltd., Wuhan, China) were performed to observe the structural changes in the skin tissues, and the expression levels of platelet endothelial cell adhesion molecule-1 (CD31) and vascular endothelial growth factor (VEGF-A) were detected using immunohistochemical staining. The mean optical density was analysed using Image-J software.

#### 2.4.3. Expression Levels of Key Genes Involved in Wound Healing

The skin tissues were removed from the wounds and stored in a refrigerator at −80 °C after being quickly frozen with liquid nitrogen on days 7 and 14. The skin tissues were ground by adding RNA extraction solution, centrifuged to obtain the supernatant, extracted with trichloromethane(Sinopharm Chemical Reagent Co., Ltd., Beijing, China) and centrifuged again to obtain the supernatant, supplemented with isopropanol(Sinopharm Chemical Reagent Co., Ltd., Beijing, China) for precipitation, centrifuged to obtain the precipitate, and then washed to dissolve the RNA. The RNA was reverse-transcribed via PCR with the temperature set at 25 °C for 5 min and then heated to 42 °C for 30 min and finally to 80 °C for 5 s. The expression levels of key genes involved in wound healing in the mouse skin tissues at the wound site were examined using fluorescence quantitative PCR (CFX Connect, Bio-rad, Hercules, CA, USA). The primers used are listed in Table 1.

### 2.5. Statistical Analysis

One-way analysis of variance (ANOVA) was applied to the results obtained using GraphPad Prism 10 to analyse the differences between the groups. Differences between groups were considered significant when *p* < 0.05 (*), *p* < 0.01 (**), or *p* < 0.001 (***), while differences were considered remarkably statistically significant when *p* < 0.0001 (****).

## 3. Results

### 3.1. Preparation and Characterisation of BATC Composite Membrane

#### 3.1.1. BATC Composite Membrane Structure

**Figure 1:**
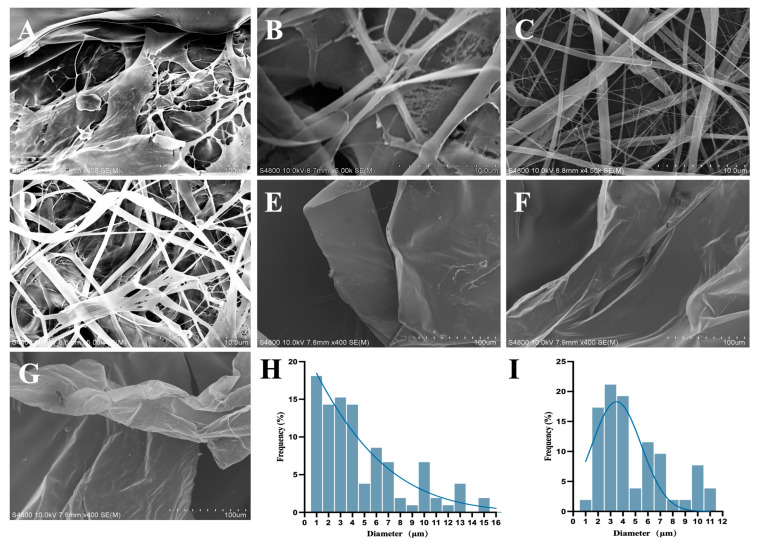
SEM images of EBP with different ratios: (**A**) 20:1, (**B**) 10:1, and (**C**) 5:1; (**D**) SEM image of EBPK; SEM images of BATC freeze-dried composite membrane: (**E**) FB, (**F**) FBP, and (**G**) FBPK; (**H**) fibre diameter distribution of EBP; (**I**) fibre diameter distribution of EBPK.

EBP at different ratios exhibited different morphologies under scanning electron microscopy, and a continuous fibre could not be obtained at a BATC-to-PEO ratio of 20:1, with such fibres exhibiting a lamellar structure. This is because clogging at the nozzle is likely to occur when spinning with this ratio, leading to the formation of large droplets on the receiver and ultimately a failure to obtain a filamentary structure (Figure 1A). With a BATC-to-PEO ratio of 10:1, only a few fibres appeared, exhibiting a mostly lamellar structure, as the membrane obtained on the receiver was not homogeneous when spun at this ratio (Figure 1B). When the ratio of BATC to PEO was 5:1, the spinning efficiency was higher, the filament output was better, and a fibrous membrane with a more desirable structure could be obtained, indicating that increasing the PEO concentration increases spinning efficiency and leads to fibres that are more uniform and fine (Figure 1C). Since we aimed to use a larger proportion of BATC in this experiment and use as little PEO as possible to achieve spinning in the subsequent experiments, a spinning solution with a BATC:PEO ratio of 5:1 was selected. We added 0.2% KuB to the spinning solution with a BATC-to-PEO ratio of 5:1, and EBPK was obtained, the morphology of which is shown in Figure 1D. There is no structural difference in the morphologies of FB, FBP, and FBPK, which have the same compositional ratio as EBP, and all of them are lamellar or laminar structures with a macroscopic, thin, sponge-like appearance (Figure 1E–G).

#### 3.1.2. Fourier-Transform Infrared Spectroscopy Analysis

**Figure 2:**
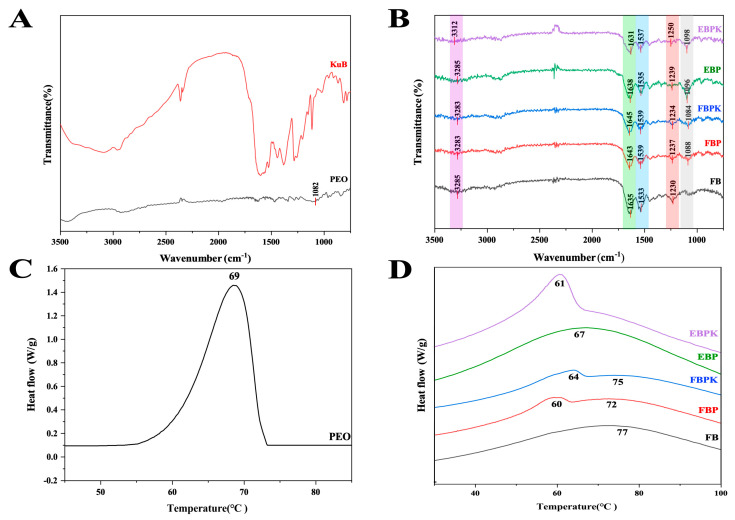
FTIR spectra of BATC composite membranes: (**A**) PEO and KuB and (**B**) BATC electrospun composite membrane and BATC freeze-dried composite membrane; DSC spectra of BATC composite membranes: (**C**) PEO and (**D**) BATC electrospun composite membrane and BATC freeze-dried composite membrane.

FTIR allowed us to detect the compositions of functional groups and observe the effect of interactions between collagen and PEO and collagen and KuB on the characteristic peaks. The characteristic absorption peaks of PEO and KuB were determined using the IR spectrogram in Figure 2A. As shown in Figure 2B, the addition of PEO to the two composite membranes increased the absorption peaks near 1082 cm^−1^, which were formed by the C-O-C stretching vibrations in PEO, indicating that PEO was successfully incorporated into the materials. Additionally, the peaks at amide A and amides I, II, and III were basically unmoved, indicating that the addition of PEO did not destroy the triple-helix structure of collagen. The absorption peak at 3285 cm^−1^ shifted to 3312 cm^−1^ after the addition of KuB to the electrospun composite membrane, a shift due to hydrogen bonding between KuB and collagen, whereas there was no hydrogen bonding in the freeze-dried membrane. As shown in Figure 2B, the absorption peaks at 3285 cm^−1^, 1635 cm^−1^, 1533 cm^−1^, and 1230 cm^−1^ are characteristic absorption peaks of collagen. The absorption peak at 3285 cm^−1^ is that of the amide A band, generated by the stretching vibration of the N-H bond. The absorption peak at 1635 cm^−1^ for the amide I band was generated by the stretching vibration of the C=O bond. The absorption peak at 1533 cm^−1^ for the amide II band was generated by the coupling of the bending vibration of the N-H bond and the stretching vibration of the C-N bond. The absorption peak at 1230 cm^−1^ for the amide III band was generated by the stretching vibration of the amide bond C-N and the bending vibration of the backbone N-H bond as well as the rocking vibration of CH_2_ on the peptide chain backbone and in proline [24].

#### 3.1.3. Thermal Stability Analysis

DSC analysis was carried out to investigate the denaturation temperature of the BATC composite membranes. As shown in Figure 2C, the thermal denaturation temperature of PEO is 69 °C. As shown in Figure 2D, the thermal denaturation temperature of EBP reduced to 67 °C due to the complexation of PEO. The addition of KuB changed the thermal absorption peak of EBPK to 61 °C, and this peak fused with another collagen heat absorption peak at a slightly higher temperature. As shown in Figure 2D, the thermal denaturation temperature of FB was 77 °C. FBP had two heat absorption peaks at 60 °C and 72 °C since the PEOs in EBP were more homogeneously dispersed than those in FBP. The addition of KuB increased the temperatures of the two heat absorption peaks of FBPK, resulting in improved thermal stability.

#### 3.1.4. Antioxidant Activity

**Figure 3:**
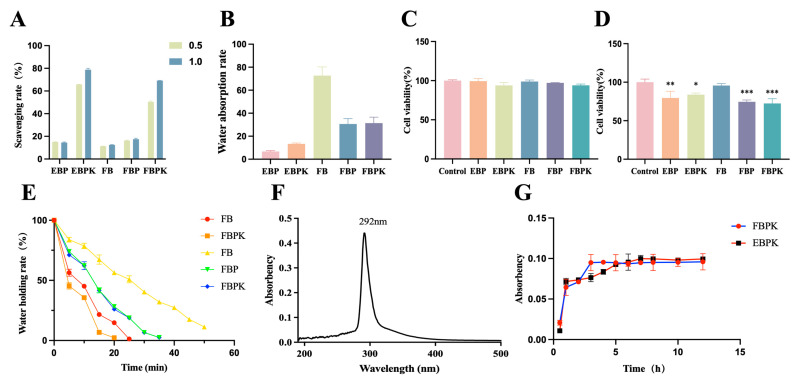
(**A**) Comparison of DPPH free-radical-scavenging capacity of BATC composite membranes at different time points; (**B**) water absorption rate of BATC composite membranes; the viability of HSF cells (**C**) treated with BATC composite membrane extract and (**D**) grown on BATC composite membrane scaffolds was detected by the CCK-8 method; (**E**) water holding rate of BATC composite membranes; (**F**) UV absorption spectrum of KuB; (**G**) in vitro drug release curve of EBPK and FBPK (* indicating *p* < 0.05, ** indicating *p* < 0.01, and *** indicating *p* < 0.001).

The antioxidant activity of the composite membranes was measured by analysing the DPPH free-radical-scavenging capacity. Figure 3A shows that the free-radical-scavenging rates of EBP and FBP slightly increased after the addition of PEO, reaching as high as 15% to 20%. KuB has a strong radical-scavenging ability, so the addition of KuB to a composite membrane can greatly improve the scavenging ability of DPPH radicals. The DPPH radical-scavenging rate of EBPK reached 65.73% at 0.5 h and then 78.79% at 1 h. The DPPH radical-scavenging rate of FBPK reached 50.39% at 0.5 h and then 69.16% at 1 h, possibly because the surface area of the electrospun composite membrane is greater than that of the freeze-dried composite membrane with the same mass and KuB has a stronger radical-scavenging effect.

#### 3.1.5. Absorbency and Retention Capacity

A good water absorption capacity allows a wound dressing to effectively absorb exudate, but too much water absorption will produce a pumping effect in the wound, which is not conducive to wound healing, so a BATC electrospun composite membrane is more suitable as a wound dressing [25]. The data in Figure 3B reveal that collagen has a good water absorption capacity, and the water absorption rate of FB reached 72.65. The water absorption rate decreased after the addition of PEO, amounting to 6.77 for EBP and 30.65 for FBP. The addition of KuB slightly improved the water absorption rate because KuB is rich in hydrophilic groups such as hydroxyl groups. Specifically, the water absorption rate increased to 13.36 for EBPK and 31.37 for FBPK. Various methods reported for the preparation of composite membranes based on BATC, such as electrospinning techniques (e.g., DCB-composite nanofibrous scaffolds) and freeze-drying techniques (e.g., C/GP/Co hydrogel) [26,27], have indicated the ability to optimise the physicochemical properties of the materials, such as hydrophilicity.

The moderate water retention of the BATC composite membrane ensures that a wound is moist whilst allowing excess exudate to evaporate. The changes in the water retention of the BATC composite membrane placed in the oven at 37 °C for different lengths of time are shown in Figure 3E, showing that the BATC freeze-dried membrane had good water retention, and the addition of both PEO and KuB reduced the water retention capacity of collagen, which could retain water in the oven at 37 °C for 30 min. The water retention capacity of the BATC electrospun composite membrane was slightly lower than that of the BATC freeze-dried composite membrane, and all the water evaporated after 20 min.

#### 3.1.6. Kukoamine B Release In Vitro

The sustained release of drugs in biomedical materials helps to stabilise the onset of a drug’s action, maintain its effective concentration, prolong the duration of a drug’s action, and reduce the frequency of administration. In order to evaluate the ability of the composite membranes to maintain and regulate the sustained release of the drug in question, the in vitro cumulative release of KuB was monitored for 12 h. As shown in Figure 3F, the UV absorption peak of KuB was at 292 nm, so 292 nm was used to detect the amount of KuB while plotting the drug release curves. As shown in Figure 3G, EBPK was released at a rapid rate within 1 h, and the release was essentially over by 6 h. The release of FBPK ended at 3 h, and the release curve converged to a horizontal straight line, suggesting that electrospun composite membranes can release KuB at a rate slower than that of freeze-dried composite membranes.

### 3.2. Cytotoxicity and Cytocompatibility

The toxicity of a dressing in direct contact with a wound or skin tissue is also an important indicator of the safety of the response. The viability of HSF cells treated with the BATC composite membrane extract was detected via CCK-8; as shown in Figure 3C, there is no significant difference between the five samples compared to the control, indicating that none of the five extracts are cytotoxic. The viability of HSF cells grown on BATC composite membrane scaffolds was detected via CCK-8, as shown in Figure 3D. The cell growth of FB was not significantly different from that of the control. Additionally, the viabilities of the cells on EBP, EBPK, FBP, and FBPK were all in the range of 70–80%, indicating that these conditions hardly affected the normal growth of the cells, and the slightly lower viability than that of the control group might be due to the fact that the level of cell adhesion for the composite membrane was slightly lower than that of the cell culture plate. A large number of studies exist showing the good cytocompatibility of BATC composite membranes. For example, IONP-CM (iron oxide nanoparticle-modified collagen membrane) did not show cytotoxicity [28].

### 3.3. Evaluation of Wound-Healing Ability

#### 3.3.1. Wound Area Evaluation

**Figure 4:**
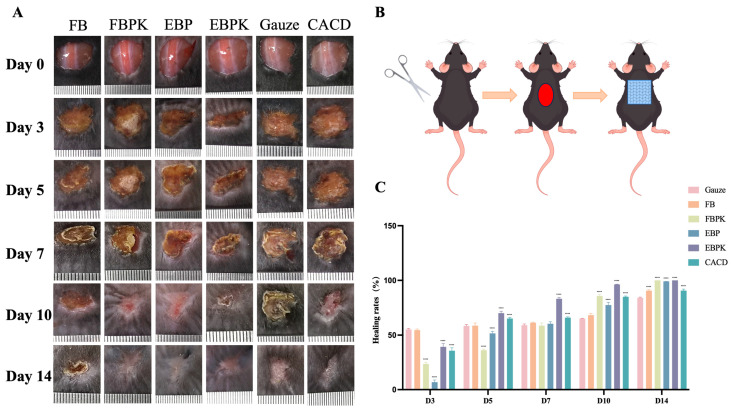
(**A**) The effect of different dressings on wound healing in mice; (**B**) mice treatment; (**C**) wound-healing rate of mice under the action of different dressings (* indicating *p* < 0.05, ** indicating *p* < 0.01, *** indicating *p* < 0.001, and **** indicating *p* < 0.0001).

To investigate the promotive effect of the prepared composite membrane on animal skin wound healing, we established a full-thickness skin injury model using C57BL/6 mice. The wounds of the mice treated with different dressings showed a tendency to heal on day 3, with a gradual reduction in the wound area, and they were almost completely healed by day 14 (Figure 4A). Wound contraction started on day 3, and the FB and gauze groups healed similarly and better than the other groups, suggesting that a better rate of healing can be achieved through the direct use of collagen dressings and gauze during the inflammatory response period in the pre-healing phase of wound healing. On day 5, the EBPK group had significantly less eschar than the other groups; on day 10, the eschar had basically fallen off, the wounds of the EBPK group healed significantly better than those of the other groups, and the wounds of the FBPK group also healed significantly better than those of the gauze control group; and on day 14, the wounds had basically healed, and they were flatter and smoother in the FBPK, EBP, and EBPK groups.

On days 7 and 10, the wound-healing rate in the EBPK group was significantly higher than that in the other groups, amounting to 83.22% and 96.29%, respectively (Figure 4C). On days 10 and 14, every other group had a significantly higher wound-healing rate than the gauze group. After day 7, all other groups exhibited better wound healing with the collagen dressing than the gauze group. This is because collagen provides good wound support and modulates some of the growth factors promoting wound healing. EBPK was the most effective at promoting wound healing because of the addition of KuB, which can act as an antioxidant, and FBPK was also superior to FB and commercial collagen dressings in the later stages of healing. A rat tendon injury model in some studies has shown that composite membranes can improve biomechanical properties [26,29], and the mouse skin injury model in this study further confirms their potential in epidermal repair.

#### 3.3.2. Histological Observation of the Wounds

We used haematoxylin–eosin (H&E) and Masson staining to observe the re-epithelialisation of tissues and collagen formation during wound repair. Haematoxylin stains chromatin in the nucleus and nucleic acids in the cytoplasm a purplish-blue colour, and eosin stains components of the cytoplasm and extracellular matrix a red colour. Masson staining dyes collagen fibres blue and myofibrils and fibrils purplish red.

**Figure 5:**
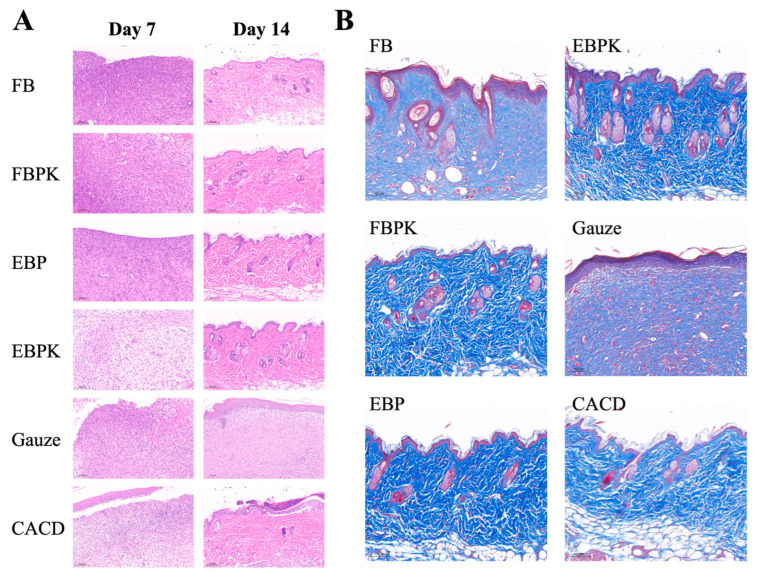
(**A**) Mouse wound skin tissue sections from different treatment groups (H&E staining); (**B**) mouse wound skin tissue sections from different treatment groups on day 14 (Masson staining).

The results of the staining of wounded skin sections from different treatment groups using H&E are shown in Figure 5A. On day 7, there was a significant inflammatory response with no epidermal layer formation at the wound site in mice from all groups. There was more neovascularisation and granulation tissue in the other five groups compared to the gauze group, with the EBPK group having the most neoangiogenesis, followed by the FBPK and CACD groups. On day 14, the re-epithelialisation of the wounds was essentially complete: a complete epidermal layer, visible as a newborn stratum corneum, had formed; dense collagen fibres were visible; and the wounds had essentially healed. Hair follicles and sebaceous glands appeared in all groups except the gauze group and the FB group, indicating that all experimental materials could promote skin neogenesis in mice. Only small quantities of stratum corneum and blood vessels formed in the gauze group, and there was also an infiltration of inflammatory cells and fewer collagen fibres, conditions different from those of normal skin. The EBPK group had the most hair follicles and sebaceous glands, with collagen fibres neatly arranged in line with normal skin; the FBPK and CACD groups also had more hair follicles and sebaceous glands but not as many as the EBPK group. It has been shown that BATC accelerates healing by promoting collagen deposition (especially collagen type I) [30,31] and angiogenesis [32,33].

The results of the Masson staining of wounded skin sections from different treatment groups on day 14 are shown in Figure 5B. On day 14, the FBPK, EBP, EBPK, and CACD groups all produced a large number of collagen fibres, which were significantly denser than those in the FB and gauze groups. Collagen deposition was neater in the FBPK, EBP, and EBPK groups, and this fact also prevented scarring due to collagen overgrowth.

#### 3.3.3. Immunohistochemical Evaluation

**Figure 6:**
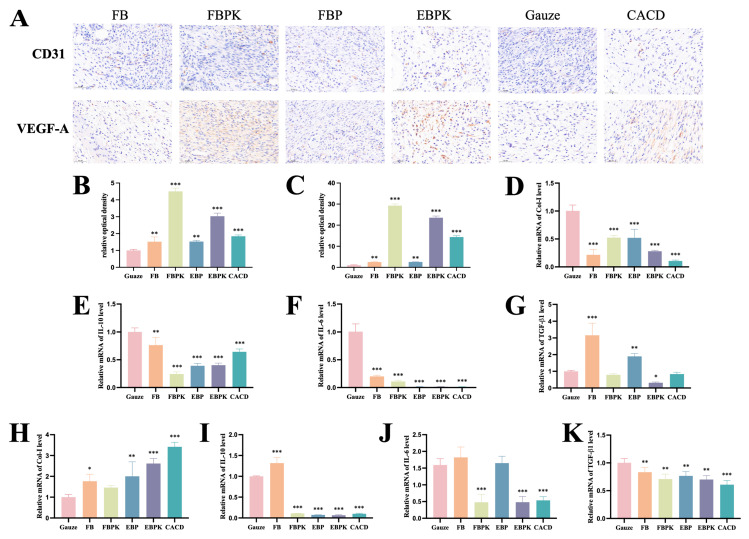
Immunohistochemical staining analysis of CD31 and VEGF-A in wounds treated by different groups on the 7th day: (**A**) immunohistochemical staining images, (**B**) relative optical density of CD31, and (**C**) relative optical density of VEGF-A. Wounded-skin-related factor mRNA expression levels of mice in different treatment groups on the 7th day: (**D**) Col-I, (**E**) IL-10, (**F**) IL-6, and (**G**) TGF-β1. Wounded-skin-related factor mRNA expression levels of mice in different treatment groups on the 14th day: (**H**) Col-I, (**I**) IL-10, (**J**) IL-6, and (**K**) TGF-β1 (* indicating *p* < 0.05, ** indicating *p* < 0.01, and *** indicating *p* < 0.001).

Neovascularisation is essential to the wound-healing process, and adequate re-establishment of blood flow ensures that sufficient oxygen and nutrients are supplied to the peri-wound tissues while also facilitating the removal of metabolic waste products, thus creating a favourable microenvironment for wound healing [34]. High expression of CD31 is one of the features of a particular subtype of blood vessel (H-vessel) that is closely associated with angiogenesis, while VEGF-A promotes neovascularisation and increases vascular permeability [35,36], so we detected VEGF and CD31 in the wound tissues using immunohistochemical staining on the 7th day of treatment, as shown in Figure 6A–C. CD31(+) and VEGF-A(+) cells were significantly upregulated in both the sample and CACD groups compared with the gauze group, suggesting that BATC composite dressings could promote angiogenesis by regulating CD31 and VEGF-A factors. The FBPK and EBPK groups had the most CD31(+) and VEGF-A(+) cells, suggesting that the promotion of angiogenesis in the FBPK and EBPK groups was optimal, consistent with the H&E staining results.

#### 3.3.4. Expression Levels of Key Genes Involved in Wound Healing

The expression levels of four key genes involved in wound healing, Col-I, IL-10, IL-6, and TGF- β1, were investigated using qRT-PCR.

The mRNA expression levels of wounded-skin-related factors in the mice in the different treatment groups on day 7 are shown in Figure 6D–G. Col-I, IL-10, and IL-6 were significantly downregulated in the sample and CACD groups compared with the levels in the gauze group. TGF-β1 was significantly upregulated in the FB and EBP groups and downregulated in the EBPK group. The significant downregulation of Col-I in the sample group may be due to the fact that some of the Col-I in the dressing could be utilised directly by the wound, so less collagen needed to be synthesised. The significant downregulation of IL-10 and IL-6 indicated that the sample group was effective in reducing the wounds’ inflammatory responses. TGF-β1 stimulates fibroblasts to support and promote wound protein synthesis, and the FB and EBP groups had significantly increased expression of TGF-β1. The EBPK group, with a wound-healing rate of 83% on day 7, was in the late stage of healing, with a decrease in TGF-β1 expression.

The mRNA expression levels of wounded-skin-related factors in the mice in the different treatment groups on day 14 are shown in Figure 6H–K. The upregulation of Col-I in comparison with the levels in the gauze group was significant for all the sample and CACD groups except for the FBPK group, suggesting that BATC dressings may promote the expression of Col-I in the later stages of healing. IL-10 was significantly upregulated in the FB group and significantly downregulated in the FBPK, EBP, EBPK, and CACD groups. IL-6 was significantly downregulated in the FBPK, EBPK, and CACD groups. These results indicate that the wound dressings supplemented with KuB were effective in reducing the inflammatory response in the late stage of healing. TGF-β1 was significantly downregulated in both the sample and CACD groups because the wounds had already healed by day 14, and the downregulation of TGF-β1 attenuated scar formation.

## 4. Discussion

Injuries, burns, contusions, cuts, trauma, diseases, and surgical incisions may inflict various wounds on the skin, and these wounds can evolve into chronic wounds if they fail to heal over time [37]. Chronic wounds are always more expensive to treat, and surgical incisions incur the highest wound-related costs, followed by diabetic foot ulcers [38], placing a more severe burden on patients and their families. And with the millions of patients with chronic wounds each year, these wounds place a significant burden on the medical system. The prevention of wound infection, proper care, and appropriate dressings are important factors in promoting wound healing [39].

Electrospun fibres offer significant advantages over other wound treatments such as hydrogels or conventional dressings. The structures of electrospun fibres are very similar to the extracellular matrix (ECM) of the skin, which allows for cell migration [40,41]. Electrostatically spun fibrous materials are one of the carriers of interest due to their structural characteristics, such as large surface area/volume ratios, flexible surface functionality, and tuneable surface morphologies. These features make it easy to tailor drug delivery to the target region, and the drug release profile can be modulated by adjusting the morphology, porosity, and composition of the fibres [42].

Collagen, a major component of the ECM, is essential for the mechanical protection of tissues and organs as well as the physiological regulation of the cellular environment, and it is widely used in biomedical and pharmaceutical applications [43]. Collagen is more biocompatible and less antigenic than other natural polymers. It is also a surfactant that penetrates lipid-free interfaces [44]. Venugopal et al. [45] incorporated collagen in electrospun nanofibre membranes to increase their porosity and improve their mechanical properties. There are now more primary processed meat products, and deep processing is becoming more backward. One byproduct of cattle slaughter, the Achilles tendon, has not been fully utilised, even though the collagen content of the Achilles tendon is up to 20% or more. The extraction of collagen from the Achilles tendon and deep processing to improve the value of the product would have a far-reaching impact on the realisation of sustainable development. Bovine Achilles tendon is rich in collagen, mainly type I collagen, which has the same collagen structure as human skin and is more favourable for human absorption. Compared with aquatic animal collagen, Bovine Achilles Tendon Collagen (BATC) has a higher content of sub-amino acids, which makes its collagen triple helix more stable [46], and BATC has superior thermal stability [47]. In this study, two forms of collagen dressings, a freeze-dried composite membrane and electrospun composite membrane, were prepared, and an in-depth comparison of the physicochemical properties and wound-healing promoting effects of the two dressings was carried out.

To promote wound healing, many studies are now functionalising dressings with plant-derived compounds to create novel biomaterials capable of accelerating the skin wound-healing process. Loading natural polymers with bioactive substances such as polysaccharides, terpenoids, alkaloids, saponins, essential oils, fatty acids, and polyphenols can stimulate the healing process because these natural molecules have anti-inflammatory, antioxidant, and antibacterial properties [48]. Compared to more commonly used dressings loaded with more cytotoxic silver nanoparticles [49] or shorter-half-life rhEGF [50], KuB has lower toxicity, has greater anti-inflammation and proangiogenesis properties, and is less expensive; therefore, KuB was used as a candidate for loading drugs.

The KuB-loaded BATC composite membrane developed in this study provides physical support through collagen scaffolds and simultaneously regulates the inflammatory microenvironment by using KuB, reflecting the synergistic strategy of “biomimetic repair + active regulation”. While it inherits the benefits of existing collagen-based dressings, it shows better healing potential through the synergistic effect of natural drugs and process innovation.

However, there are some limitations of this study; for example, in terms of drug release control, although the composite membrane can achieve a slow release of the drug, the release kinetics and long-term stability still need to be optimised to avoid sudden release or premature depletion. Moreover, although electrospun membranes can mimic the extracellular matrix, the random fibre arrangement may not be sufficient to guide the directional growth of tendon cells, and more precise bionic structures need to be developed (e.g., Aligned-to-Random bilayer membranes) [51]. Dynamic release systems could be optimised in the future, e.g., by investigating responsive release mechanisms (e.g., pH- or enzyme-triggered) to match the molecular requirements at different stages of tissue repair [26,52] and exploring coaxial electrospinning techniques to encapsulate multi-drugs (e.g., antibiotics + growth factors) [53,54]. Multidisciplinary techniques can be integrated, such as combining 3D printing and microfluidics to optimise the spatial structure of membranes [55] or using transcriptome analysis (e.g., TNF/NF-κB pathway) to guide material design [51,56].

## 5. Conclusions

In this study, a BATC electrospun composite membrane containing 16.7% PEO and 0.2% KuB and a BATC freeze-dried composite membrane were prepared using electrospinning technology and freeze-drying technology to investigate the biocompatibility and wound-healing effects of the dressings. The characterisation results for the BATC composite membrane showed that it has good physicochemical properties, and we expect that it will be applied as a dressing to promote wound healing. The electrospun formulation has better water absorption properties and achieves a slow release of the composite drug for a sustained antioxidant effect. The results of cellular experiments showed that the BATC composite membrane was non-cytotoxic and had little effect on cellular activity. Experiments conducted using a mouse full-thickness skin injury model showed that the BATC composite membrane promoted skin wound healing, especially in the middle of the healing process, in which EBPK promoted the fastest skin wound healing. In conclusion, the BATC composite membrane can promote wound healing in mice, and owing to the excellent physical properties granted by the electrospinning process and the antioxidant effect of KuB, it endows EBPK, which can be intensively researched and developed as a biomaterial, with the best healing-promotive effect.

## Figures and Tables

**Table 1:** Primer sequence information table.

Gene	Primer	Primer Sequences
Gapdh	Forward	CAAGCTCATTTCCTGGTATGACAAT
	Reverse	GTTGGGATAGGGCCTCTCTTG
Col-I	Forward	TCCTGACGCATGGCCAAGAAGACA
	Reverse	TCCGGGCAGAAAGCACAGCACTC
IL-10	Forward	GCTCCAAGACCAAGGTGTCTACAA
	Reverse	CCGTTAGCTAAGATCCCTGGATCA
IL-6	Forward	TCCAGTTGCCTTCTTGGGAC
	Reverse	GCCATTGCACAACTCTTTTCTCA
TGF-β1	Forward	CAACAATTCCTGGCGTTACCTTGG
	Reverse	GAAAGCCCTGTATTCCGTCTCCTT

## Data Availability

The original contributions presented in this study are included in the article. Further inquiries can be directed to the corresponding authors.
